# Rate and key predictors of unplanned readmission following ischemic stroke: a systematic review and meta-analysis

**DOI:** 10.3389/fneur.2026.1776757

**Published:** 2026-05-20

**Authors:** Ting Zhang, Yingchun Li, Jianrong Du, Qiurun Zhong, Xuefang Yang, Wenqin Yang, Huan Wang

**Affiliations:** 1 College of Nursing, Jinzhou Medical University, Jinzhou, China; 2 Department of Nursing, The Second People’s Hospital of Yibin, Yibin, China; 3 Department of Gynecology, The Second People’s Hospital of Yibin, Yibin, China; 4 Department of Neurosurgery, The Second People’s Hospital of Yibin, Yibin, China; 5 Department of Information Technology, The Second People’s Hospital of Yibin, Yibin, China

**Keywords:** evidence-based nursing, ischemic stroke, meta-analysis, rate, risk factors, unplanned readmission

## Abstract

**Objective:**

To systematically evaluate the rate and risk factors for unplanned readmission in patients with ischemic stroke (IS), providing an evidence-based foundation for developing targeted prevention strategies.

**Methods:**

We systematically searched PubMed, Web of Science, Embase, Cochrane Library, CNKI, Wanfang, Weipu Data and SinoMed databases for studies on the rate and risk factors of unplanned readmission in IS patients from their inception to July 30, 2025. Two researchers independently conducted literature screening, quality assessment, and data extraction. Meta-analysis was performed.

**Results:**

A total of 44 studies involving 1,274,267 patients were included. The pooled rate of 30-day readmission was 14.2% (95% CI 11–18%), increasing to 13.9% at 90 days and 30.3% at 12 months, with substantial heterogeneity across studies (*I*^2^ > 90%). Readmission rates were lower in North America (11.7%) and higher in Asian settings (17.2%). Atrial fibrillation (OR 1.34), heart failure (OR 1.59), diabetes (OR 1.29), and prior stroke (OR 1.50) were associated with increased likelihood of 30-day readmission. Longer hospital stay (OR 1.03 per unit increase) and greater stroke severity (NIHSS OR 1.13) were also associated with higher readmission risk. Infection (25.1%), recurrent stroke (21.8%), and cardiac events (13.7%) were the most consistently reported causes.

**Conclusion:**

This meta-analysis clarifies and updates the rate and core risk factors for unplanned readmission in ischemic stroke patients. It provides an evidence-based foundation for systematically identifying high-risk patients, developing risk assessment tools, and implementing targeted interventions, holding significant value for guiding clinical practice.

**Systematic review registration:**

https://www.crd.york.ac.uk/PROSPERO/view/CRD420251157352, identifier CRD420251157352.

## Introduction

Ischemic stroke (IS) represents a leading cause of mortality and long-term disability worldwide, contributing to a significant and growing public health burden (1, 2). In the continuum of stroke care, the period following hospital discharge is critical, with unplanned readmissions emerging as a pivotal indicator of healthcare quality and system performance (3). These readmissions not only signify potential gaps in transitional care and suboptimal patient management but are also strongly associated with poorer patient outcomes, diminished quality of life, and substantial economic costs (4, 5). Consequently, the precise identification of individuals at elevated risk and a clear understanding of the determinants of readmission are fundamental to developing effective prevention strategies and optimizing post-stroke care pathways.

While the clinical and economic significance of unplanned readmissions is well established, the existing synthesized evidence base is constrained by notable inconsistencies, methodological limitations, and a critical temporal gap, which collectively limit its practical utility for clinicians and policymakers. Previous systematic reviews and meta-analyses have laid important groundwork by consolidating data on readmission rate and risk factors in stroke populations (6–9). However, these efforts are inherently limited as their literature searches concluded by 2021 or earlier (6–9), thereby excluding a substantial and growing body of recent research conducted amidst significant evolution in stroke management protocols and healthcare policies.

Beyond this timeliness issue, the evidence base is characterized by substantial and unresolved heterogeneity. Reported rates vary puzzlingly across studies, ranging from single-digit percentages to over 30–40% even within similar timeframes (8, 9). While potential sources of this variation, such as differences in healthcare system structures, study designs (e.g., prospective vs. retrospective), and definitions of the readmission window, are often acknowledged, they have rarely been the subject of rigorous, pre-specified subgroup meta-analyses capable of explaining this variance and generating context-specific estimates (9). Consequently, clinicians and health system managers lack clear, applicable benchmarks for their own settings. Furthermore, the evidence regarding key risk factors requires consolidation and clarification. Prior reviews have listed numerous potential predictors, but an updated synthesis is needed to distinguish robust, independent risk factors validated across diverse settings from those with inconsistent, weak, or context-dependent associations, which may be susceptible to confounding or influenced by study design (6, 8). The relative importance of established factors (e.g., stroke severity) versus emerging or less-studied variables also necessitates recalibration with contemporary data.

To address these gaps, we conducted an updated systematic review and meta-analysis. This study aimed to estimate the global pooled rate of unplanned readmission following ischemic stroke by incorporating evidence published up to date. We seek to provide a refined and current evidence base that can inform the development of more accurate risk prediction models, support the implementation of personalized, patient-centered interventions during hospitalization and beyond, and offer actionable insights for health policy. This study aimed at improving care transitions and reducing preventable readmissions among patients with ischemic stroke on a global scale.

## Materials and methods

### Literature search strategy

We systematically searched PubMed, Web of Science, Embase, Cochrane Library, CNKI, Wanfang, Weipu Dta, and SinoMed. Additionally, Baidu Scholar and Google Scholar were searched to supplement grey literature. Searches employed a combination of subject headings and free-text terms, covering the period from database inception to July 30, 2025. The search strategy was designed to maximize sensitivity, employing a combination of controlled vocabulary (e.g., MeSH terms) and free-text keywords. The core search concepts included terms related to ischemic stroke (e.g., “stroke,” “cerebrovascular accident,” “brain infarction”) and unplanned readmission (e.g., “patient readmission,” “rehospitalization,” “hospital readmission”). The detailed search strategy, including the specific databases searched and the complete syntax for all search strings, is provided in the Supplementary material.

### Inclusion and exclusion criteria

Studies were eligible for inclusion if they met the following criteria: the population consisted of adult patients (≥18 years) with a clinical diagnosis of ischemic stroke; the study reported data on the rate of unplanned readmission (within any timeframe, such as 30 days, 90 days, or 1 year) and/or its associated risk factors or prediction models; and the study design was observational, including cohort and case–control studies. Studies were excluded if they were narrative reviews, case reports, or conference abstracts; if the full text was unavailable or essential data were incomplete; or if the study focused on specific ischemic stroke subpopulations not representative of the general adult patient cohort, such as patients in special physiological states like pregnancy.

### Literature screening and data extraction

Literature screening was performed independently by two investigators according to the pre-defined inclusion and exclusion criteria. The process involved two stages: an initial review of titles and abstracts, followed by a full-text assessment of potentially eligible records. Any disagreements between the two reviewers were resolved through discussion or, if necessary, by consulting a third senior researcher to reach a consensus. Data extraction from the included studies was conducted independently by the same two investigators using a standardized, piloted data extraction form. Extracted information included basic study characteristics (first author, publication year, country), methodology (study design), population details (sample size, inclusion criteria), definitions and metrics related to unplanned readmission (follow-up timeframe, readmission rate), and outcome data [reported risk factors and their corresponding effect sizes, such as Odds Ratios (OR) with 95% Confidence Intervals (CI)]. The extracted data were then cross-checked, and any discrepancies were discussed and resolved to ensure accuracy.

### Literature quality assessment

The quality of included observational studies was assessed using the Newcastle–Ottawa Scale (NOS) (10). Quality assessment was conducted independently by two researchers and cross-checked. Any discrepancies were resolved through discussion or adjudication by a third researcher.

### Statistical analysis

As the risk of readmission is inherently dependent on the duration of follow up, and definitions of readmission varied across studies, we defined 30-day readmission as the primary outcome *a priori*. Readmission at 90 days and 1 year were analysed as secondary outcomes and were examined separately.

NoteExpress 4.0 software was used for literature management. Statistical analyses were conducted using Stata version 15.0 (StataCorp) and R version 4.4.1 (R Foundation for Statistical Computing). Readmission rate was pooled as proportions with corresponding 95% confidence intervals. Heterogeneity was assessed using the Cochrane *Q* test and *I*^2^ statistic. A fixed-effects model was used if *I*^2^ ≤ 50% and *p* ≥ 0.10; otherwise, a random-effects model was employed.

Effect estimates of individual risk factors were extracted as reported, including odds ratios with corresponding confidence intervals. Where effect estimates were not provided, but sufficient data were available, crude odds ratios were derived from reported event counts. Given differences in reporting across studies, pooling was undertaken only when effect measures and exposure definitions were considered sufficiently comparable. Continuous and categorical representations of the same variable were analysed separately. For variables reported using heterogeneous categorical definitions or differing thresholds, quantitative synthesis was not performed and findings were summarised narratively to preserve clinical interpretability. All pooled estimates were interpreted as associations rather than causal effects.

To explore potential sources of heterogeneity, subgroup analyses were conducted according to country, study design, and data source.

Publication bias was examined using funnel plots and Egger’s test. Sensitivity analysis was performed using the leave-one-out method to test result stability. All analyses were two-sided, with a significance level of *α* = 0.05.

## Results

### Literature search and screening results

**Figure 1:**
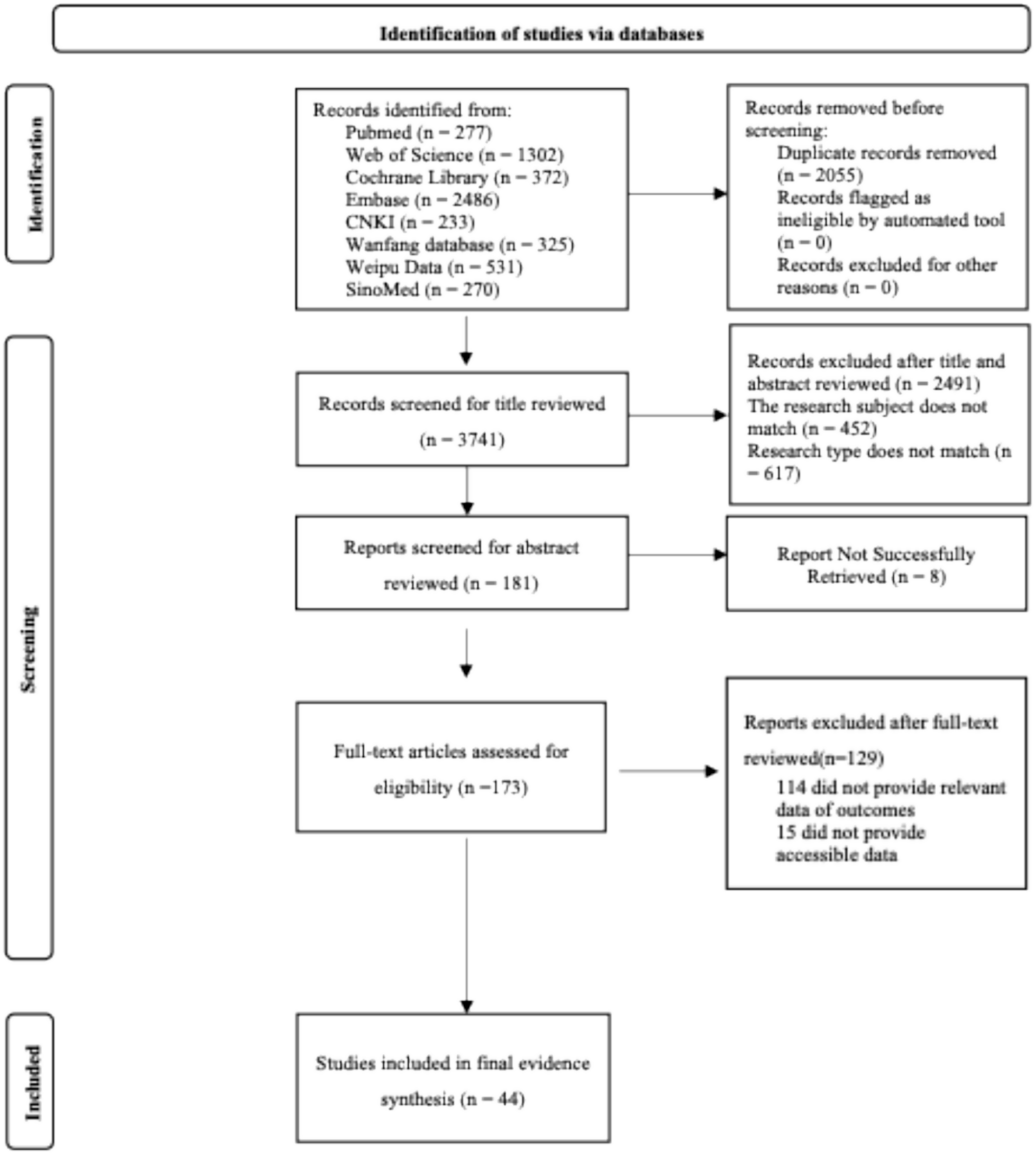
Literature screening flow diagram following PRISMA 2020.

The initial search yielded 5,796 records. After removing duplicates, 3,741 remained. Following title/abstract screening and full-text review, 44 studies were ultimately included for quantitative synthesis. The literature screening process is detailed in Figure 1.

### Basic characteristics and quality assessment of included studies

The 44 included studies involved 1,274,267 patients, with publication years ranging from 2001 to 2025 cross 8 countries. Twenty studies (44.4%) were published in the recent 5 years (2020–2025). Study subjects came from various countries. The primary study types were retrospective (35 studies) and prospective (8 studies), with one case–control study. The definition of unplanned readmission was mostly within 31 days, with some studies extending it to 90 days or 1 year. Quality assessment indicated 9 studies were of high quality, with the remainder being of moderate quality. The characteristics and quality assessment results of the included studies are presented in Table 1.

**Table 1:** Characteristics and quality assessment of included studies.

First author	Country	Study type	Unplanned readmission	Risk factors	NOS score^a^
Jun-O'Connell et al. (17)	USA	Retrospective	30 days	①②③④	6
Anna et al. (18)	Norway	Prospective	90 days; 1 year	⑤⑥⑦	5
Keyhani et al. (19)	USA	Retrospective	30 days	⑤⑦⑧⑨⑩⑪	6
Hu et al. (20)	China	Prospective	30 days	⑤⑪⑫⑬⑭⑮	7
Xu et al. (21)	China	Retrospective	90 days	⑤⑧⑬	6
Nouh et al. (22)	USA	Case–control	30 days	⑤⑦⑪⑲	6
Tay (23)	Singapore	Retrospective	1 year	⑤⑮	7
Al Sibani et al. (24)	Oman	Retrospective	28 days	⑤⑦⑮⑰	6
Kilkenny et al. (25)	Australia	Retrospective	30 days	⑥⑯⑰	6
Leitao et al. (26)	Portugal	Retrospective	1 year	⑪	6
Qiu et al. (27)	China	Retrospective	30 days	⑤⑦⑪⑯⑱	5
Lily et al. (28)	USA	Retrospective	30 days; 1 year	⑤⑥⑦⑧⑮⑲	6
Lin et al. (29)	Taiwan, China	Prospective	1 year	⑤⑦⑮⑯	5
Wen et al. (30)	China	Retrospective	31 days	⑤⑮⑱⑲	6
Ang et al. (31)	Malaysia	Retrospective	28 days	⑤⑥⑮	6
Anna et al. 2018 (32)	Norway	Prospective	30 days	⑤⑥⑮㉒	5
Bjerkreim et al. (33)	Norway	Prospective	1 year	⑥⑦㉓	6
Liu et al. (34)	China	Retrospective	30 days	⑤⑥⑦⑨⑮⑲⑳	6
Ma et al. (35)	China	Retrospective	31 days	⑦⑭⑮㉓㉔㉕	5
Bhattacharya et al. (36)	USA	Retrospective	30 days	⑪	7
Mittal et al. (37)	USA	Retrospective	30 days	③⑤⑥⑦㉓	6
Lv et al. (38)	China	Retrospective	30 days	⑪	7
Bohannon and Lee (39)	USA	Retrospective	30 days	②⑤⑥⑪⑮⑱	6
El Naamani et al. (40)	USA	Retrospective	30 days	㉕	6
Nakagawa et al. (41)	USA	Retrospective	30 days	⑤⑥⑨	5
Cun et al. (42)	China	Retrospective	30 days	⑪⑮㉓	6
Shah et al. (43)	USA	Prospective	30 days	⑪	7
Kilkenny et al. (44)	Australia	Retrospective	31 days	⑥⑦⑱⑳	7
Lee et al. (45)	South Korea	Retrospective	30 days	①⑤⑥⑮⑲⑳	5
Chen et al. (46)	USA	Prospective	30 days	⑤⑥⑫⑭⑮⑱	8
Lineback et al. (47)	Australia	Retrospective	30 days	⑦	7
Li et al. (48)	Taiwan, China	Retrospective	30 days; 6 months; 1 year	②③⑤⑦⑱	5
Nkoke et al. (49)	Cameroon	Prospective	1 year	–	6
Abreu et al. (50)	Portugal	Retrospective	1 year	⑤⑥⑦⑪⑮㉓㉕	6
Man et al. (51)	USA	Retrospective	30 days	⑤⑥⑧㉓㉕	5
Fehnel et al. (52)	USA	Retrospective	30 days	⑦㉒㉓	6
Darabi et al. (53)	USA	Retrospective	30 days	⑪	6
Slocum et al. (54)	USA	Retrospective	30 days	–	6
Miao (55)	China	Retrospective	30 days	⑤⑦⑮㉔㉕	8
Wen et al. (56)	China	Retrospective	31 days	⑥⑮⑲⑳㉑	5
Gao et al. (57)	China	Retrospective	31 days	⑤⑮㉓	5
Benjamin et al. (12)	China	Retrospective	31 days	③④⑦⑬⑮㉓㉕	6
Lu et al. (58)	China	Retrospective	90 days	⑤⑧⑪㉔	6
Xu et al. 2018 (59)	China	Retrospective	90 days	⑮ ㉓	5

① Place of residence; ② Barthel index; ③ Marital status; ④ Dyslipidemia; ⑤ Age; ⑥ Gender; ⑦ Comorbidities; ⑧ Low income; ⑨ Psychiatric illness; ⑩ APACHE score; ⑪ NIHSS; ⑫ BMI; ⑬ ADL score; ⑭ Education level; ⑮ Length of hospital stay; ⑯ History of stroke; ⑰ Charlson comorbidity index score; ⑱ Diabetes mellitus; ⑲ Medical insurance; ⑳ Hospital level; ㉑ Occupation; ㉒ Enteral nutrition; ㉓ Hypertension; ㉔ Hcy; ㉕ Smoking.

a NOS, Newcastle–Ottawa Scale (range 0–9), used to assess the quality of included observational studies; higher scores indicate better methodological quality.

### Unplanned readmission rate in IS patients

**Figure 2:**
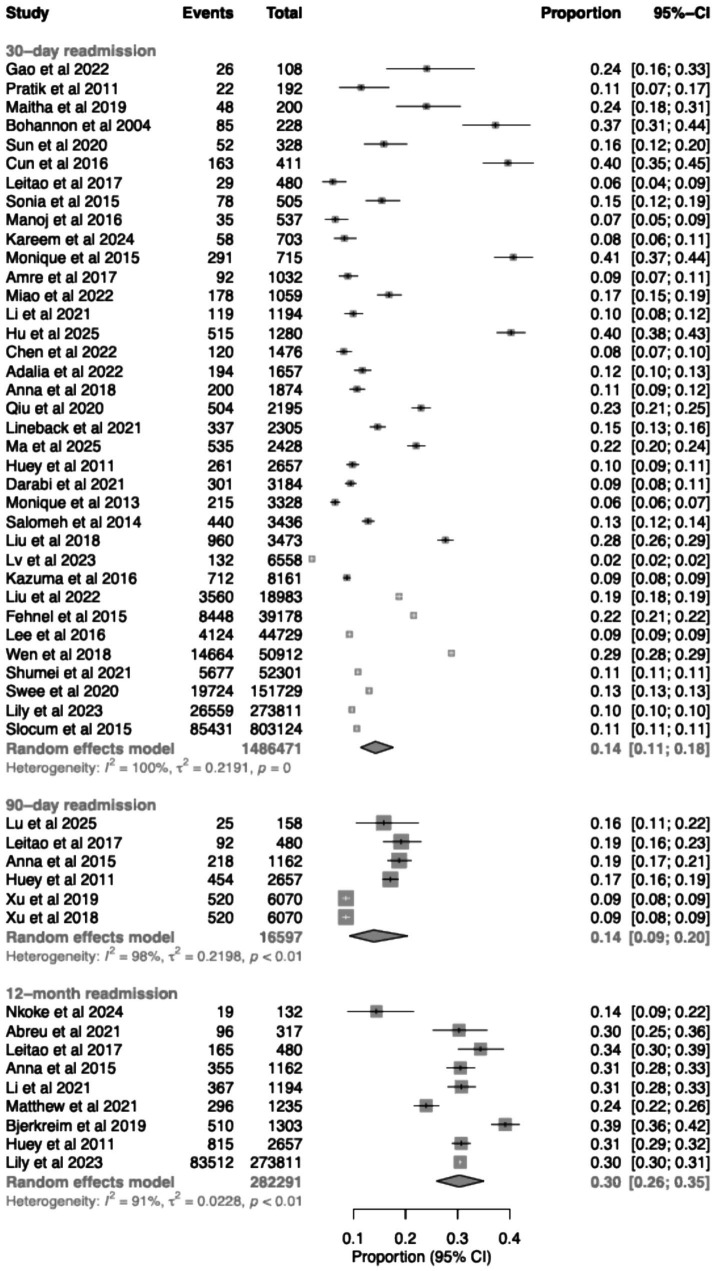
Forest plots of unplanned readmission rates at 30 days, 90 days, and 12 months following ischemic stroke.

A total of 44 studies were included in the analysis. Among 36 studies reporting 30-day readmission, the pooled rate was 14.2% (95% CI 11.4–17.7%, *I*^2^ = 99.8%) (Figure 2). Six studies reported 90-day readmission, with a pooled rate of 13.9% (95% CI 9.2–20.4%, *I*^2^ = 98.1%). Nine studies reported 12-month readmission, with a pooled rate of 30.3% (95% CI 25.9–35.1%, *I*^2^ = 90.9%).

When stratified by country, variation in readmission rate was observed across regions (Supplementary Figure S1a). The pooled estimate was 11.7% (95% CI 9.0–14.9%) in North America and 17.2% (95% CI 11.4–25.3%) in Asian settings. Estimates for Europe and Western Pacific region showed wide confidence intervals. Norway [10.7% (95% CI 9.4–12.2%)] and Portugal [6.0% (95% CI 4.2–8.6%)] were the only countries representing European settings, each contributing a single study. Australia contributed three studies, with a pooled estimate of 16.7% (95% CI 1.1–78.0%), although the wide confidence interval reflects substantial uncertainty. Australia, representing the Western Pacific region, contributed three studies, with a pooled estimate of 16.7% (95% CI 1.1–78.0%). Africa was represented by a single study with an estimated rate of 24.0% (95% CI 18.6–30.4%).

When stratified by data source, studies conducted in tertiary hospital settings reported a pooled rate of 15.5% (95% CI 11.1–21.1%), compared with 12.3% (95% CI 9.4–15.9%) for studies based on administrative or database sources (Supplementary Figure S1b).

Stratification by study period demonstrated some variation in readmission rate over time (Supplementary Figure S1c). The pooled estimate was 9.8% (95% CI 6.0–15.6%) for studies published between 2010 and 2014, 15.9% (95% CI 10.6–23.2%) for studies from 2015 to 2019, and 13.2% (95% CI 9.4–18.2%) for studies published between 2020 and 2025. A single study contributed to the period before 2010, reporting a higher rate of 37.3%.

### Risk factors for unplanned readmission in IS patients

**Figure 3:**
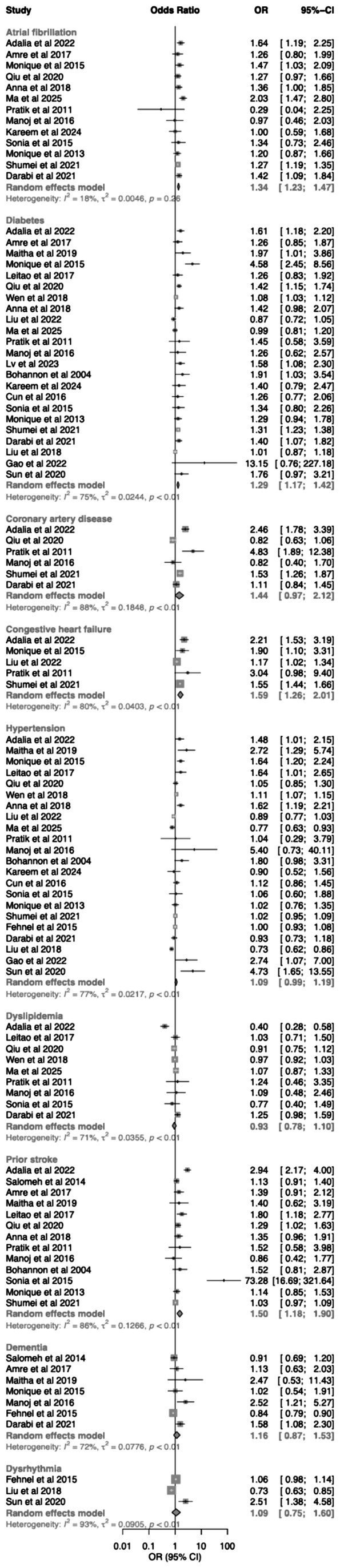
Forest plot showing pooled associations between comorbid conditions and 30-day readmission.

The association between comorbid conditions and 30-day readmission was examined across multiple studies (Figure 3). Atrial fibrillation was associated with an increased likelihood of readmission, with a pooled odds ratio of 1.34 (95% CI 1.23–1.47) based on 13 studies, with relatively low heterogeneity (*I*^2^ = 18.4%). Similarly, diabetes mellitus was associated with a higher readmission risk (OR 1.29, 95% CI 1.17–1.42; 23 studies), although heterogeneity was moderate (*I*^2^ = 74.6%). Congestive heart failure and prior stroke were also associated with increased readmission risk. The pooled odds ratio was 1.59 (95% CI 1.26–2.01; 5 studies) for congestive heart failure and 1.50 (95% CI 1.18–1.90; 13 studies) for prior stroke. In contrast, coronary artery disease showed a similar direction of association but did not reach statistical significance (OR 1.44, 95% CI 0.97–2.12; 6 studies), with substantial heterogeneity observed. Hypertension was associated with a modest increase in risk that did not reach statistical significance (OR 1.09, 95% CI 0.99–1.19; 22 studies). Dyslipidemia showed no evidence of association with readmission (OR 0.93, 95% CI 0.78–1.10; 9 studies). Dementia and dysrhythmia were not significantly associated with readmission.

Stroke severity measures were associated with higher 30-day readmission risk. Greater neurological deficit, as measured by NIHSS, was associated with increased readmission (OR 1.13, 95% CI 1.01–1.26); results are shown in Supplementary Figure S2.

Length of hospital stay was an important risk factor. Three studies reported length of hospital stay as a continuous variable, expressed as odds ratios per unit increase (Supplementary Figure S3a). A pooled analysis showed that longer hospital stay was associated with an increased likelihood of 30-day readmission (OR 1.03, 95% CI 1.00–1.06). Although the magnitude of effect was modest, the direction of association was consistent across studies. Given the small number of included studies, this finding should be interpreted with caution. Another five studies (Supplementary Figure S3b) examined the association between length of hospital stay and 30-day readmission using categorical definitions, with substantial variation in thresholds and reference groups. Given this heterogeneity, direct comparison across studies is limited. A pooled estimate was not performed, and results are presented in Supplementary Figure S3b.

### Causes of readmission

**Figure 4:**
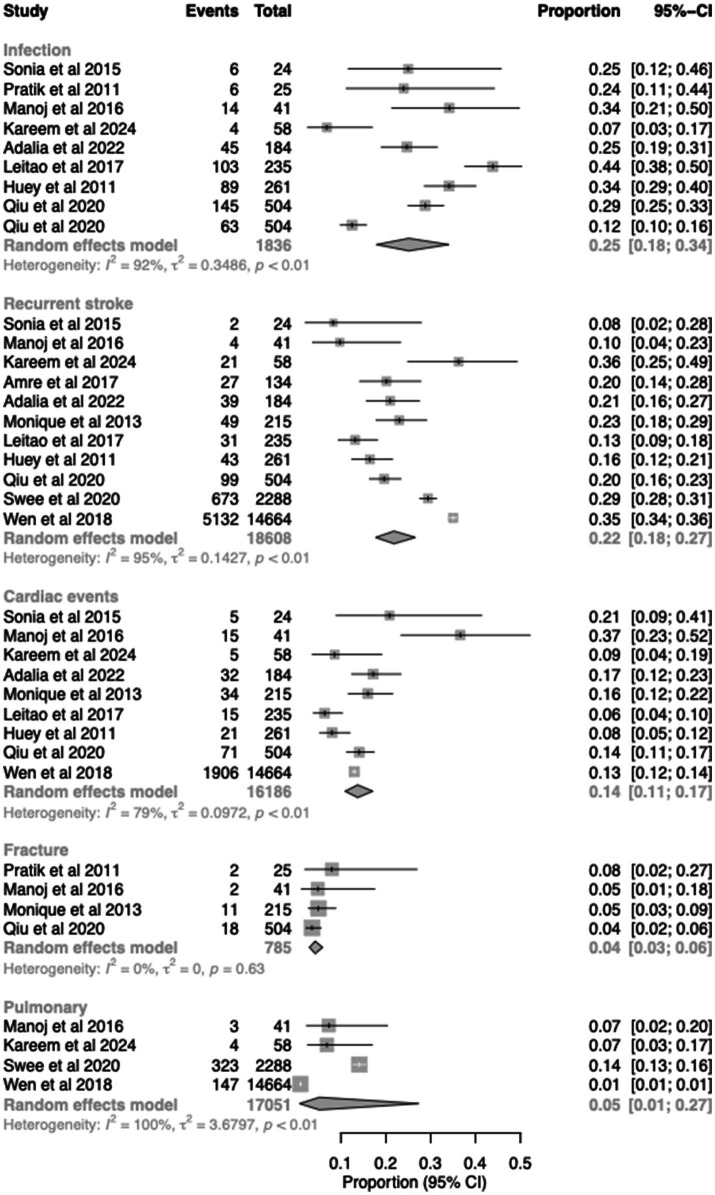
Forest plot for causes of readmission.

Causes of 30-day readmission were heterogeneous across studies (Figure 4, Supplementary Table S1). Infection (25.1, 95% CI 18.0–34.0%), recurrent stroke (21.8, 95% CI 17.7–26.6%), and cardiac events (13.7, 95% CI 11.0–17.1%) were the most consistently reported causes. Pulmonary causes, excluding pulmonary infections, (5.0, 95% CI 1.0–27.0%) and fractures (4.3, 95% CI 3.0–6.0%) were less frequently reported. Substantial heterogeneity was observed across most categories, and additional causes are summarised in Supplementary Table S1.

### Sensitivity analysis and publication bias

Funnel plot (Supplementary Figure S4) asymmetry was assessed using Egger’s test, which showed no evidence of small-study effects (*p* = 0.20). However, given the substantial between-study heterogeneity, this finding should be interpreted with caution.

Leave-one-out analyses (Supplementary Figure S5) demonstrated that exclusion of any single study did not materially change the pooled estimate (range 13.7–14.9%), suggesting that the findings were not driven by any individual study. Heterogeneity remained persistently high across all iterations, indicating that between-study variability reflects systematic differences rather than the influence of outlying studies.

## Discussion

This systematic review and meta-analysis found that approximately 14% experienced readmission within 30 days following ischemic stroke, indicating that early readmission is a common and clinically important event. Although the risk remained similar at 90 days, it increased to nearly one third by 1 year, suggesting that vulnerability to adverse outcomes persists well beyond the immediate post-discharge period. Notably, the causes of readmission were not limited to recurrent stroke but were frequently driven by infections and cardiac complications, highlighting that post-stroke recovery involves a broad range of medical risks. The association of readmission with comorbid conditions such as atrial fibrillation, heart failure, diabetes, and prior stroke further underscores the contribution of underlying disease burden. In addition, factors such as longer hospital stay and greater stroke severity were associated with increased readmission risk, likely reflecting clinical complexity rather than direct causal effects. These findings suggest that the risk of readmission after stroke is sustained and multifactoria.

Our pooled estimate of 30-day readmission (approximately 14%) is broadly consistent with prior reports, including the 17.4% reported by Zhong et al. (8), although somewhat higher than the 10.66% observed by Deng et al. (6). While the substantial heterogeneity warrants cautious interpretation of the overall estimate, the subgroup analyses provide insight into potential sources of variation. In particular, differences across regions were observed, with lower readmission rates in North America (approximately 12%) and higher rates in Asian settings (approximately 17%). Estimates from other regions were less precise, reflecting limited data. Evidence from European settings was especially sparse, with only single studies from Norway (11%) and Portugal (6%), which precludes meaningful comparison and suggests that apparent differences should be interpreted cautiously. These disparities likely reflect fundamental differences in healthcare system structures, the accessibility and integration of post-discharge rehabilitation services, and the influence of national health policies. For instance, financial incentive programs like the U.S. Hospital Readmissions Reduction Program, which penalizes hospitals for excess readmissions, may have driven systematic efforts to improve transitional care, contributing to lower observed rates (11). Second, readmission risk demonstrated a clear temporal pattern, accumulating significantly over time. The rate 1 year (30%) was nearly double that within 30 days (14%). This finding strongly argues for extending the clinical and quality monitoring focus beyond the conventional 30-day window, advocating for care continuity models that support patients for at least 1 year post-discharge.

Several clinical characteristics were associated with an increased likelihood of 30-day readmission following ischemic stroke, most notably atrial fibrillation, heart failure, diabetes, and prior stroke. These findings are consistent with existing literature demonstrating that patients with a higher burden of cardiovascular comorbidity remain at elevated risk of early complications and recurrent events after stroke. Atrial fibrillation, in particular, is a well-established risk factor for recurrent ischemic stroke and systemic embolism, even in the context of anticoagulation, and has been associated with increased early readmission and mortality in observational cohorts (12, 13). Similarly, heart failure reflects impaired cardiac function and haemodynamic instability, which may predispose patients to both recurrent vascular events and non-neurological complications requiring rehospitalization (14).

Diabetes and prior stroke were also associated with increased readmission risk, likely reflecting cumulative vascular injury and increased susceptibility to both recurrent cerebrovascular events and systemic complications. Prior studies have shown that diabetes is associated with poorer functional recovery and higher risk of infection and cardiovascular events after stroke, while a history of prior stroke indicates advanced cerebrovascular disease and residual disability (14). In contrast, hypertension and dyslipidaemia showed weaker or non-significant associations, which may reflect their high prevalence and widespread treatment in stroke populations, reducing their discriminatory value as predictors of early readmission.

Importantly, these factors should not be interpreted as direct causal determinants of readmission. Rather, they represent markers of underlying disease burden and patient vulnerability. The observed associations likely reflect a combination of increased baseline risk, greater clinical complexity, and higher susceptibility to post-discharge complications. This interpretation is supported by the finding that readmission causes were frequently non-neurological, including infections and cardiac complications, suggesting that early readmission is driven by multisystem instability rather than recurrent stroke alone.

At the health policy level, the marked international variation in readmission rates provides valuable lessons. Countries with higher rates may need to evaluate and strengthen the linkages between hospital care, post-acute rehabilitation, and primary care services. Conversely, strategies employed in lower-rate settings, such as value-based payment models that incentivize reduced readmissions, demonstrate that policy levers can influence system behavior, though their broader impacts require careful evaluation (15, 16). Furthermore, the strong time-dependent increase in risk challenges the adequacy of the 30-day readmission metric as a sole measure of care quality. Policymakers and health systems are encouraged to adopt a more longitudinal view, potentially incorporating 90-day or 1-year readmission measures into performance frameworks to encourage sustained patient support and improve long-term outcomes (16).

This study has several limitations. First, substantial heterogeneity was observed across studies, reflecting differences in patient populations, healthcare systems, outcome definitions, and analytical approaches. Although subgroup analyses were performed, much of this variability remained unexplained. Second, variation in reporting and model adjustment across studies may affect comparability of effect estimates. While adjusted estimates were preferentially used, some crude odds ratios were derived from reported data, which may contribute to residual confounding. Third, variability in the definition and reporting of readmission causes limited the ability to perform a fully standardised synthesis. Although causes were grouped into clinically meaningful categories, differences across studies may affect interpretation.

The associations identified in this study should be interpreted as reflecting patient vulnerability and clinical complexity rather than direct causal relationships. Future research should focus on clarifying causal pathways and validating these factors in prospective studies. Establishing causal relationships will be essential before translating these findings into robust clinical prediction tools and targeted intervention strategies. In addition, incorporating broader clinical, social, and behavioral determinants may improve risk stratification and support more personalised approaches to post-stroke care.

## Conclusion

This meta-analysis provides an updated and comprehensive evidence base on the rate and determinants of unplanned readmission after ischemic stroke. It confirms a significant global burden and identifies key modifiable and non-modifiable risk factors, including stroke severity, atrial fibrillation, diabetes, and length of hospital stay. These findings offer crucial guidance for clinicians to identify high-risk patients, for health systems to design targeted interventions spanning the acute to chronic care continuum, and for policymakers to develop more nuanced quality metrics. Future work should focus on translating these factors into usable risk prediction models and integrating broader social and behavioral determinants to advance the field from generalized to precise, personalized prevention strategies.

## Data Availability

The raw data supporting the conclusions of this article will be made available by the authors, without undue reservation.
