# Supplementary material for: Rate and key predictors of unplanned readmission following ischemic stroke: a systematic review and meta-analysis
Source: Front Neurol. 2026 May 20;17:1776757. doi: 10.3389/fneur.2026.1776757 (PMC13232766; doi:10.3389/fneur.2026.1776757)
Supplement: Supplementary file 1 [file Table_1.docx]

Table S1 Cause of readmission

| study | year | cause | event | readmission |
| --- | --- | --- | --- | --- |
| Leitao et al | 2017 | Extracranial hemorrhage | 14 | 235 |
| Qiu et al | 2020 | Sepsis | 5 | 504 |
| Qiu et al | 2020 | Seizures | 36 | 504 |
| Qiu et al | 2020 | TIA | 16 | 504 |
| Qiu et al | 2020 | Venous thromboembolism | 18 | 504 |
| Huey et al | 2011 | Neurologic sequelae of stroke | 28 | 261 |
| Huey et al | 2011 | Ulcer disease with bleeding | 15 | 261 |
| Wen et al | 2018 | Hypertension | 0 | 14664 |
| Wen et al | 2018 | Diabetes | 0 | 14664 |
| Wen et al | 2018 | Mental or neurologic disorder | 0 | 14664 |
| Wen et al | 2018 | Spinal disorder | 0 | 14664 |
| Swee et al | 2020 | Sepsis | 0 | 2288 |
| Pratik et al | 2011 | metabolic derangements | 3 | 25 |
| Pratik et al | 2011 | hematemesis | 1 | 25 |
| Pratik et al | 2011 | hypotension | 1 | 25 |
| Manoj et al | 2016 | Seizure | 1 | 41 |
| Kareem et al | 2024 | Seizures | 2 | 58 |
| Monique et al | 2013 | Neurological and mental disorders | 0 | 215 |
| Monique et al | 2013 | Cardinal manifestation | 0 | 215 |
| Monique et al | 2013 | Respiratory disease | 0 | 215 |

Figure S1a. Forest plots for subgroup analyses by country

Figure S1b. Forest plots for subgroup analyses by data source

Figure S1c. Forest plots for subgroup analyses by publication year

Figure S2. Forest plot of the association between stroke severity and 30-day readmission following ischemic stroke.

Figure S3a. Forest plot of the association between LOS (continuous) and 30-day readmission following ischemic stroke.

Figure S3b. Forest plot of the association between LOS (categorical) and 30-day readmission following ischemic stroke.

Figure S4. Funnel plot

Figure S5. Leaving one out analysis
